# Beyond Pathogenic *RUNX1* Germline Variants: The Spectrum of Somatic Alterations in RUNX1-Familial Platelet Disorder with Predisposition to Hematologic Malignancies

**DOI:** 10.3390/cancers14143431

**Published:** 2022-07-14

**Authors:** Alisa Förster, Melanie Decker, Brigitte Schlegelberger, Tim Ripperger

**Affiliations:** Department of Human Genetics, Hannover Medical School, Carl-Neuberg-Str. 1, 30625 Hannover, Germany; foerster.alisa@mh-hannover.de (A.F.); decker.melanie@mh-hannover.de (M.D.); schlegelberger.brigitte@mh-hannover.de (B.S.)

**Keywords:** *RUNX1* germline variants, RUNX1-FPD, leukemia predisposition, hematologic malignancies, somatic mutations

## Abstract

**Simple Summary:**

Pathogenic germline variants affecting *RUNX1* are associated with qualitative and/or quantitative platelet defects, and predispose to hematologic malignancies. The latter manifests in approximately 44% of carriers and can occur from early childhood to late adulthood. In addition to the predisposing *RUNX1* germline variant, the acquisition of somatic genetic alterations is presumed to drive leukemic transformation in an inflammatory bone marrow niche. The spectrum of somatic mutations occurs heterogeneously between individuals, even within families, and there is no clear genotype–phenotype correlation. In this review, we summarize previously published patients harboring (likely) pathogenic *RUNX1* germline alterations in whom somatic alterations were additionally analyzed. We provide an overview of their phenotypes and the most frequent somatic genetic alterations.

**Abstract:**

Pathogenic loss-of-function *RUNX1* germline variants cause autosomal dominantly-inherited familial platelet disorder with predisposition to hematologic malignancies (RUNX1-FPD). RUNX1-FPD is characterized by incomplete penetrance and a broad spectrum of clinical phenotypes, even within affected families. Heterozygous *RUNX1* germline variants set the basis for leukemogenesis, but, on their own, they are not transformation-sufficient. Somatically acquired secondary events targeting *RUNX1* and/or other hematologic malignancy-associated genes finally lead to MDS, AML, and rarely other hematologic malignancies including lymphoid diseases. The acquisition of different somatic variants is a possible explanation for the variable penetrance and clinical heterogeneity seen in RUNX1-FPD. However, individual effects of secondary variants are not yet fully understood. Here, we review 91 cases of RUNX1-FPD patients who predominantly harbor somatic variants in genes such as *RUNX1*, *TET2*, *ASXL1*, *BCOR*, *PHF6*, *SRSF2*, *NRAS*, and *DNMT3A*. These cases illustrate the importance of secondary events in the development and progression of RUNX1-FPD-associated hematologic malignancies. The leukemia-driving interplay of predisposing germline variants and acquired variants remain to be elucidated to better understand clonal evolution and malignant transformation and finally allow risk-adapted surveillance and targeted therapeutic measures to prevent leukemia.

## 1. Introduction

Over the past decades, it has become evident that the RUNX family transcription factor 1 (RUNX1) is a key player in embryogenesis and hematopoiesis [1]. RUNX1 is encoded by on the long arm of chromosome 21 (i.e., 21q22.12). It was previously also known as acute myeloid leukemia 1 *(AML1)*, core-binding factor A2 *(CBFA2)*, and Runt-related transcription factor 1. Three major protein isoforms of RUNX1 are known (i.e., RUNX1a, RUNX1b, and RUNX1c). Their expression is regulated by two different promoters [2,3]. The distal P1 promotor initiates the generation of transcription variant 1, which is translated to isoform RUNX1c [3]. The proximal P2 promotor and an alternative splicing mechanism drive the expression of transcription variant 2 and 3, encoding for isoform RUNX1b and RUNX1a, respectively [2]. All RUNX1 isoforms can form heterodimers with core-binding factor beta (CBFB). As a core-binding factor complex, they function as transcriptional regulators [4]. Binding to CBFB significantly enhances the DNA-binding ability of RUNX1 [5,6] and protects RUNX1 from ubiquitin-mediated proteasomal degradation [7]. The best-studied function of RUNX1 is the activation of transcription of its target genes [5,8]. Through its interplay with many cofactors and interaction partners, RUNX1 can also lead to the repression of transcription [9,10]. The essential role of RUNX1 in stem cell differentiation, especially in hematopoiesis, is highlighted by the absence of definitive hematopoietic stem cells in homozygous *Runx1* knock-out mice and their hemorrhagic death at day E.12.5 of development [11,12,13,14]. RUNX1 is involved in the differentiation of lymphoid and myeloid lineage cells, especially in the megakaryocytic lineage. Moreover, RUNX1 promotes gene expression for megakaryocyte development, while genes important for erythropoiesis are suppressed [11,15].

Somatic *RUNX1* aberrations are recurrently detected in various myeloid malignancies, such as myelodysplastic syndrome (MDS), acute myeloid leukemia (AML) [16], and myeloproliferative neoplasms. Characteristic *RUNX1* translocations (i.e., t(12;21), t(8;21), and t(3;21)), are considered as common events in hematologic malignancies (HM) [17]. To date, about 70 chromosomal translocations encompassing *RUNX1* have been reported in patients with HM [18,19]. Noteworthy, acquired mono- or biallelic somatic *RUNX1* variants, including deletions, missense, splice site, frameshift, and nonsense variants, correlate with worse prognosis in sporadic AML, MDS, and T-cell acute lymphoblastic leukemia (T-ALL) [20,21,22,23]. Therefore, AML with somatic *RUNX1* variants is considered a biologically distinct AML subtype associated with poor outcomes in the 2016 revision of the World Health Organization (WHO) classification of myeloid neoplasms and acute leukemia [24].

Regarding germline mutations, *RUNX1* was first associated with leukemia predisposition in 1999 [25]. Nowadays, pathogenic germline loss-of-function *RUNX1* variants are known to be causative of autosomal dominantly inherited familial platelet disorder with a predisposition to hematologic malignancies (RUNX1-FPD, FPDMM, FPD/AML, ORPHA: 71290, MIM: 601399), also recognized in the 2016 WHO classification of myeloid neoplasms and acute leukemia [24]. Patients with RUNX1-FPD often suffer from mild to moderate thrombocytopenia and/or platelet aggregation defects [25,26,27]. Remarkably, about 44% of individuals will develop HM, usually MDS or AML [28]. The age of onset of HM ranges from 6 to 76 years with an average age at diagnosis of 33 years [29,30]. Notably, several groups identified (likely) pathogenic germline *RUNX1* variants in up to 3% of AML patients [31,32,33]. Others reported about 8% of germline *RUNX1*-mutated cases in their AML study cohorts, although they were not solely (likely) pathogenic [34]. Future investigations based on proper germline material and variant classification are required to elucidate the impact of (likely) pathogenic *RUNX1* variants on sporadically appearing HM. In general, the incidence of (likely) pathogenic germline *RUNX1* variants might be underestimated due to no or mild non-malignant symptoms and/or late disease onset. Additionally, *RUNX1* copy number alterations (CNAs) are not always properly investigated in routine diagnostics and some next-generation sequencing (NGS) approaches are hampered by insufficient coverage of *RUNX1*. To date, more than 200 families with RUNX1-FPD have been reported [35]. However, the penetrance is incomplete and the expressivity is variable as the spectrum of clinical phenotypes, even within families, is broad [36,37]. *RUNX1* germline variants are classified as either (likely) benign, variant of uncertain significance (VUS), or (likely) pathogenic based on specific guidelines proposed by the ClinGen Myeloid Malignancy Variant Curation Expert Panel (MM-VCEP) [38], which was recently updated (https://www.clinicalgenome.org/affiliation/50034/, accessed on 7 June 2022). Yet, regarding these guidelines, many *RUNX1* variants are classified as VUS hence their probable pathogenicity needs further evaluation for classification such as functional assays, including DNA binding, hetero-dimerization with CBFB and transactivation, as performed by Decker and colleagues [39,40].

As genetic analyses have evolved from single-gene testing to NGS, different HM-associated and candidate genes can be simultaneously investigated. These advantages have not only led to the application of NGS in the identification of germline predispositions but, moreover, its implementation in analyzing the somatic mutation profile of blood and bone marrow. Somatically acquired mutations reported in HM-patients with germline *RUNX1* variants are suspected to be the cause of clonal transformation [15,41,42]. For example, Gaidzik et al. reported germline and somatic *RUNX1* variants predominantly co-occurring with a complex pattern of somatic gene mutations frequently involving mutations in epigenetic modifiers (e.g., *ASXL1*, *IDH2*, *KMT2A*, and *EZH2*), components of the spliceosome complex (e.g., *SRSF2* and *SF3B1*) and, moreover, *STAG2*, *PHF6*, and *BCOR* [43]. Additionally, acquired variants in the RAS-pathway genes (e.g., *HRAS*, *KRAS*, and *NRAS*) and other genes such as *CBL*, *CDC25C*, *FLT3*, *NFE2*, and *WT1* were described [36,44]. Loss of *RUNX1* heterozygosity and trisomy 21 with duplication of the mutant allele are also common secondary events [45]. Recently, evidence arose that the inflammatory milieu may promote progression to HM in individuals with germline susceptibility [46]. This supports the notion that germline *RUNX1* mutations are not solely sufficient to develop neoplasia, but favor the acquisition of additional somatic mutations in an inflammatory environment required for the development of overt leukemia [15]. Apart from this, the acquisition of different somatic variants may explain the variable penetrance and clinical heterogeneity seen in RUNX1-FPD. Nevertheless, understanding the clonal evolution of hematopoietic cells in germline *RUNX1*-mutated patients leading to HM remains to be elucidated [36,47].

## 2. Methods

To better define the spectrum of acquired variants in individuals with (likely) pathogenic *RUNX1* germline variants, we performed an extended systematic literature search in the PubMed database using the terms “RUNX1 germline”, “RUNX1 predisposition”, and “familial leukemia”. In this review, we initially included only patients with *RUNX1* germline variants in which somatic variants were investigated irrespective of variant findings. However, we excluded two cases in total, as these samples were not comparable to the other reviewed cases: one was described as a therapy-related AML that developed after T-ALL in a patient with the RUNX1 alteration p.(Gln335Argfs*259) [45,48] whereas the other individual harboring the *RUNX1* variant c.611G>A p.(Arg204Gln) was diagnosed with T-ALL, MDS, and secondary AML at age 22, 23, and 24, respectively [36].

If necessary, nomenclature of *RUNX1* variants was adapted to transcript variant 1 (NM_001754.4) encoding for isoform RUNX1c. All reported *RUNX1* germline variants were (re)classified using current recommendations according to the second version of the ClinGen MM-VCEP specifications of the ACMG/AMP variant interpretation guidelines [38] (https://www.clinicalgenome.org/affiliation/50034/, accessed on 7 June 2022). For further evaluation, we selected only patients with (likely) pathogenic *RUNX1* germline variants based on the current guidelines as only these were considered as confirmed RUNX1-FPD cases in the present study. Following this approach, we retrospectively enrolled 91 individuals out of 60 families reported in the original publications listed in Appendix A. To gain insights into the molecular mechanisms leading to malignant transformation and disease heterogeneity in RUNX1-FPD, we compared and evaluated the potential interplay of germline *RUNX1* variants, acquired somatic alterations and reported clinical phenotypes.

## 3. Disease-Causing *RUNX1* Germline Variants and Associated Phenotypes in RUNX1-FPD

**Figure 1:**
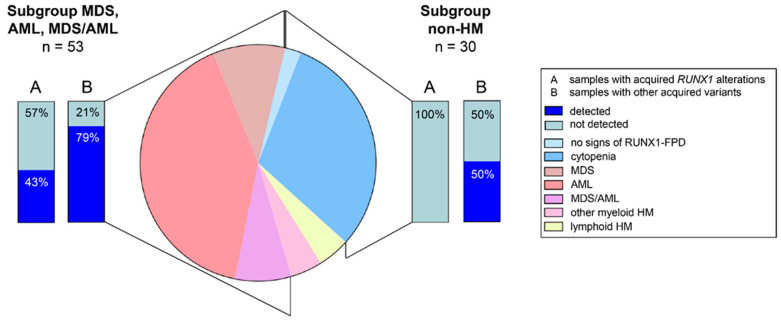
Phenotype and somatic variant status of our retrospective RUNX1-FPD cohort. Distri-bution of phenotypic subgroups within the retrospective RUNX1-FPD cohort including ratios of cases with somatic RUNX1 variants and ratios of other acquired variants. AML—acute myeloid leukemia, HM—hematologic malignancies, MDS—myelodysplastic syndrome, MDS/AML—patients who developed myelodysplastic syndrome and subsequently acute myeloid leukemia, non-HM—cases without reported hematologic malignancies, RUNX1-FPD—familial platelet disorder with predisposition to hematologic malignancies.

The integrated results of 91 included patients are given in Table 1 summarizing their clinical and genetic features. The cohort of enrolled patients mirrored the known broad phenotypic heterogeneity of patients with RUNX1-FPD. Of 91 individuals, 30 (33%) cases had no signs of HM (Figure 1). Out of these, the majority of individuals (i.e., 28, 93%) were reported with cytopenia. Two individuals (6%) had no FPD-related symptoms. This is in line with previous findings reporting thrombocytopenia as the most common phenotypic feature seen in individuals with (likely) pathogenic germline *RUNX1* variant and no HM [38]. In contrast to the previously reported HM risk of 44% [28], 61 out of the 91 enrolled patients (67%) were reported with HM. However, this high percentage is biased by our inclusion criteria focusing on patients with reported screenings for somatic alterations being a general standard in HM diagnostics but yet not in individuals with a genetic predisposition to HM. Regarding the type of malignant neoplasms, 37 individuals were diagnosed with AML (61%), nine with MDS (15%), seven with MDS/AML (11%), four with lymphoblastic HM (i.e., B- and T-ALL, 7%), and four with other myeloid malignancies (i.e., myeloproliferative neoplasm, chronic myelomonocytic leukemia, juvenile myelomonocytic leukemia, 7%) (Figure 1), which resembles the distribution of malignancy types in the RUNX1 database (RUNX1db) [35]. Recently, evidence emerged that besides T-ALL also lymphoid malignancies of B-cell origin are part of the phenotypic spectrum, even though *RUNX1* germline variants are primarily associated with myeloid malignancies [47,49]. Yet, larger cohorts are needed to evaluate this assumption.

**Figure 2:**
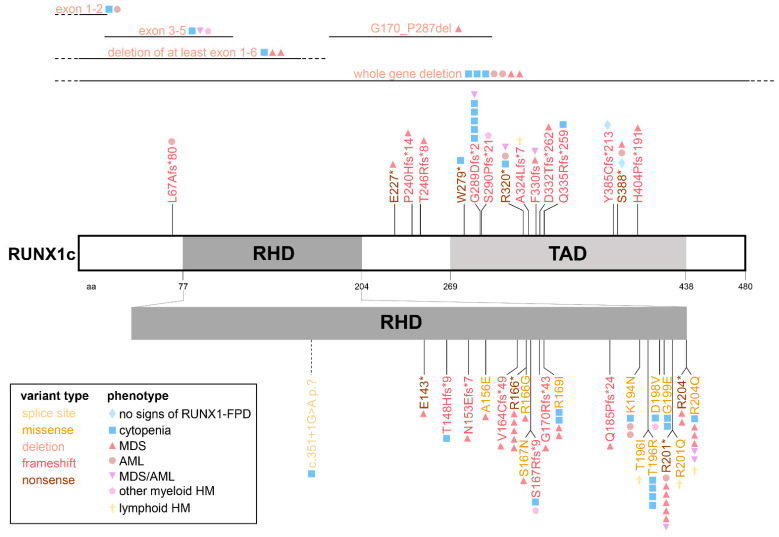
*RUNX1* germline variants in the retrospective RUNX1-FPD cohort. Schematic visualization of germline *RUNX1* variants included in the retrospective RUNX1-FPD cohort including variant type and patients’ phenotype. Nomenclature refers to transcript variant 1 (NM_001754.4) encoding for isoform RUNX1c. AML—acute myeloid leukemia, HM—hematologic malignancies, MDS—myelodysplastic syndrome, MDS/AML—patients who developed MDS and subsequently AML, RHD—runt-homology domain, RUNX1-FPD—familial platelet disorder with predisposition to hematologic malignancies, TAD—transactivation domain.

Among the herein analyzed 60 families, 17 (28%) carried frameshift variants, 15 (25%) nonsense variants, and 14 (23%) whole gene or exonic deletions. Therefore, frameshift variants, nonsense variants and deletions were found to be the most common variant types among all (likely) pathogenic germline *RUNX1* alterations. Further germline variant types comprise 13 (17%) missense variants and one (3%) splice site variant (Table 1, Figure 2). The most common disease-causing variant type in the RUNX1db are missense variants and deletions, followed by *RUNX1* frameshift and nonsense variants [35]. Noteworthy, Homan et al. reported (likely) pathogenic splice site variants in 11% of RUNX1-FPD families, whereas only one splice site variant could be included in the present study. This difference in the occurrence of *RUNX1* germline splice site variants indicates that the analyzed cohort underlies a certain bias. All 27 germline *RUNX1* missense variants are located within the Runt homology domain (RHD, amino acid 77-204), whereas nonsense and frameshift variants are evenly distributed over the gene (Figure 2), which is in line with published studies investigating type and location of germline and somatic *RUNX1* variants [36,50,51]. Additionally, all identified (likely) pathogenic germline *RUNX1* frameshift variants are unique to the affected families in the RUNX1db [35] and in the herein analyzed cohort. Other germline variant types occur in more than one individual and/or family. Arg166, Arg201 and Arg204 are the most frequently mutated RUNX1 amino acid (aa) positions in RUNX1-FPD in the RUNX1db [35] as well as in the present cohort (Figure 2). Among 30 individuals without HM, germline *RUNX1* missense and nonsense variants were reported in 37% and 10% of cases, respectively. In contrast, patients with MDS, AML or MDS/AML had missense and nonsense variants in 23% and 38% of cases, respectively. These findings may suggest that nonsense rather than missense germline *RUNX1* variants might promote progression to MDS and/or AML in the analyzed cohort. However, Brown and colleagues did not find a significant correlation between the type of variant and the risk of HM by analyzing 82 *RUNX1* germline variants [36]. Previous studies suggested that germline *RUNX1* variants causing dominant-negative effects rather than variants leading to haploinsufficiency might more potently drive the malignant transformation towards HM [27,52,53]. This hypothesis could not be supported by the analyzed RUNX1-FPD patients herein. *RUNX1* haploinsufficiency variants due to nonsense and frameshift mutations predicted to undergo nonsense-mediated decay (i.e., affecting positions before codon 304 of RUNX1c, [38]), as well as whole gene deletions, are not predominantly found in the pre-HM group of our analyses. Of note, information (i.e., functional data) on *RUNX1* missense variants regarding their dominant-negative effect is not given for most alterations. In summary, larger and unbiased cohorts are necessary to specify possible genotype-phenotype correlations in RUNX1-FPD. Available data does not justify variant type-specific risk stratifications regarding HM. The ongoing NIH Natural History Study of Hematologic and Premalignant Conditions Associated with RUNX1 Mutation will provide further insights into the natural cause of the disease and may show correlations between variant type and HM risk, as *RUNX1* germline carriers are intensively monitored over time independent of their clinical phenotype (study number 19-HG-0059, https://www.genome.gov/Current-NHGRI-Clinical-Studies/hematologic-and-premalignant-conditions-associated-with-RUNX1-mutation, accessed on 7 June 2022).

The median age of RUNX1-FPD diagnosis was highly variable (*n* = 63, median 42 years, range 0.08–74). Relevant information was not given for 28 patients (Table 1). The median age at diagnosis was lower for patients with MDS (median 33 years, range 7–58) compared to patients diagnosed with AML (median 43 years, range 0.08–74). This might illustrate the notion that in some patients, MDS might transform into AML later on. Previously, the median age at diagnosis of HM was reported as 29–35 years among *RUNX1* germline carriers [37,54]. In the RUNX1db, the median age at diagnosis is 43 (range 3–69) and for pre-leukemic patients 34 (range 1–76) [35]. In 20% of RUNX1-FPD patients, a childhood-onset malignancy was observed [37]. In line with this, our retrospective cohort encompasses 15% of cases of minors including six AML, two MDS, one B-ALL, one chronic myelomonocytic leukemia, and one juvenile myelomonocytic leukemia. Conclusively, the age of onset in our retrospective cohort and the ratio of childhood-onset cases are comparable to other RUNX1-FPD cohorts. However, it should be noted that the definition of “age at diagnosis” may vary from study to study, as some authors used this term at the time of first reported symptoms and others at the time of genetic diagnosis. Additionally, caution should be taken when comparing simplex RUNX1-FPD patients and individuals from RUNX1-FPD families, since healthy family members of index patients diagnosed by predictive testing might get earlier investigations regarding their symptoms and the presence of clonal hematopoiesis than diseased individuals without any family history of HM diagnosed due to their own phenotype.

A further limitation of the retrospective analyses in the present study was the different approaches in the included publications in order to determine the germline origin of a detected *RUNX1* variant. Authors considered variants to be of germline origin via segregation analysis, analysis of DNA derived from buccal swabs, fibroblasts, or remission material as well as approaches comparing tumor and normal tissues. When molecular profiling reveals a pathogenic variant in hematologic tissues with a variant allele fraction of at least 30% in a gene known to confer inherited cancer risk, a germline origin should be suspected and subsequently verified [55]. This and germline testing, in general, can be carried out by analysis of DNA extracted from cultured fibroblasts [56] or by segregation analyses. For the latter, however, the germline origin of the respective variant cannot be excluded if the variant is not detected in family members. Notably, genetic testing must also include analysis of copy number changes by NGS or array-CGH in order to pinpoint *RUNX1* CNAs. Taken together, we want to point out that the method (i.e., Sanger sequencing or NGS), as well as tissue (i.e., bone marrow, buccal swabs, peripheral blood, fibroblasts, fingernails), is important in terms of detection of a potential germline variant.

## 4. Spectrum of Somatic Variants and Affected Genes in RUNX1-FPD

**Figure 3:**
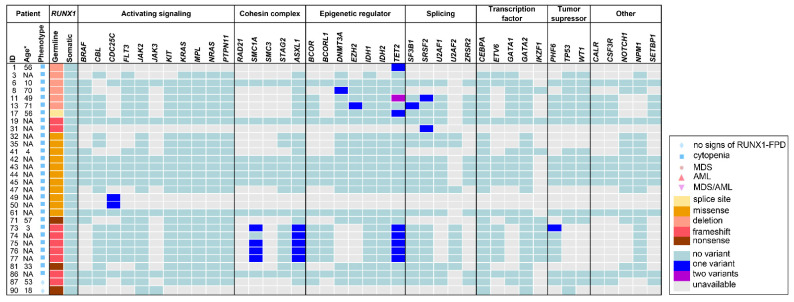
Frequently investigated and affected genes within the subgroup non-hematologic malignancy. Illustration of age, phenotype, type of *RUNX1* germline variant and analyzed somatic variants for 30 non-HM cases. For references to the individual patients please refer to the supplementary data. AML—acute myeloid leukemia, HM—hematologic malignancies, MDS—myelodysplastic syndrome, MDS/AML—patients who developed MDS and subsequently AML, NA—not available, RUNX1-FPD—familial platelet disorder with predisposition to hematologic malignancies * some authors refer to the age of first reported symptoms and others to the age of genetic diagnosis.

**Figure 4:**
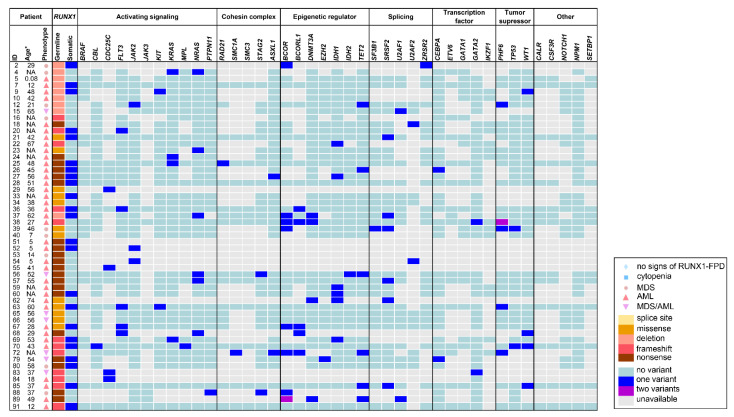
Frequently investigated and affected genes within the subgroups MDS, AML, and. MDS/AML. Illustration of age, phenotype, type of *RUNX1* germline variant and analyzed somatic variants for 53 HM cases with MDS and/or AML. For references to the individual patients please refer to the supplementary data. AML—acute myeloid leukemia, HM—hematologic malignancies, MDS—myelodysplastic syndrome, MDS/AML—patients who developed MDS and subsequently AML, NA—not available, RUNX1-FPD—familial platelet disorder with predisposition to hematologic malignancies. * some authors refer to the age of first reported symptoms and others to the age of genetic diagnosis.

Our retrospective analyses included 91 previously published individuals carrying (likely) pathogenic *RUNX1* germline variants, who were analyzed for additional somatic gene variants regardless of whether a somatic variant was identified or not. Somatic alterations were investigated by karyotyping, array-CGH, and/or DNA sequencing (i.e., mainly NGS panels). Overall, 31 of 56 (55%) cases with given karyotype information showed a somatically abnormal karyotype, all of which were associated with HM. Interestingly, of 19 patients with acquired somatic *RUNX1* mutations, 15 had an abnormal karyotype (79%). In four of these cases chromosome 21 was affected, leading to duplication of the mutated *RUNX1* allele, detected via karyotyping and variant allele fraction of the *RUNX1* germline variant. In contrast, only 14 (41%) cases were reported with an abnormal karyotype in 34 cases without somatic *RUNX1* alterations and available karyotype information. Noteworthy, *RUNX1* somatic status was not analyzed in two cases with abnormal karyotype. A summary of frequently investigated and mutated genes is given in Figure 3 and Figure 4. Additional detailed information on all detected somatic variants and analyzed genes can be found in Supplementary Table S1. Collectively, a median of 28 (range 1–51) genes was analyzed per sample (*n* = 91). Overall, *RUNX1* represents the most frequently analyzed gene (i.e., investigated in 89 of 91 cases, 98%). Other commonly analyzed genes were *GATA2*, *PTPN11*, *CEBPA*, *JAK2*, *IDH1*, *IDH2*, *KIT*, *KRAS*, *NRAS*, *NPM1*, *ASXL1*, *CBL*, and *MPL*. On a median, two (range 0–32) somatic variants were detected per sample. Among all 91 reported cases, the most common somatically altered genes were *RUNX1*, *TET2*, *ASXL1*, *BCOR*, *PHF6*, and *SRSF2*, while variants in other genes were uniquely reported in specific malignancies. Remarkably, no additional acquired variants were detected in 21 (23%) of 91 samples. These cases were primarily associated with a pre-leukemic phenotype corroborating the notion that acquisition of somatic alterations is correlated with disease progression, particularly malignant transformation. The mutational signature of the four enrolled lymphoblastic HMs differed from myeloid samples as somatic variants were recurrently found in *NOTCH1*, *PHF6*, and *TET2*. Somatic *RUNX1* alterations were found in 23 of all cases (25%) and in 23 of 53 patients (43%) with reported MDS, AML, or MDS/AML. None of the 30 non-HM patients had a secondary somatically acquired *RUNX1* variant. *RUNX1* being the most frequently investigated gene was also the most frequently mutated gene. This high frequency was significantly higher than in patients with assumed sporadic AML and is in line with data from previous reports, as somatic alterations of *RUNX1* were reported as the most common somatic mutation in patients with RUNX1-FPD (i.e., 36%) [36]. Noteworthy, the authors of studies included in our retrospective analysis did not investigate if *RUNX1* germline and somatic variants appear in *cis* or *trans*. All 23 carriers of an additional *RUNX1* somatic variant were diagnosed with MDS and/or AML. In these patients, the median number of additionally acquired variants was two variants per sample (range 0–10) with only four cases without any additionally acquired variant besides *RUNX1*. Additional alterations were frequently found in *FLT3*, *IDH1*, *SRSF2*, *WT1*, and *BCOR*. On the contrary, a median of one variant per sample (range 0–20) (e.g., in *TET2*, *ASXL1*, *SRSF2*, *PDS5B*, and *NUP214*) was detected among 66 individuals who harbored no somatic *RUNX1* variant and included 36 with and 30 without a reported HM. Of note, no somatic *RUNX1* variants were reported in subgroups with lymphoid or other myeloid HM. In summary, the most frequently mutated genes differ from those that were frequently analyzed, except for *RUNX1*. Thus, future sequencing panels need adaptation to include, at least, the most common somatically altered genes in RUNX1-FPD to improve monitoring of clonal hematopoiesis and malignant transformation in these patients. All RUNX1-FPD patients harboring somatic *RUNX1* variants were diagnosed with MDS, MDS/AML, or AML, the majority of them had clonal cytogenetic alterations and carried additional somatic alterations in genes despite *RUNX1*. This indicates that in RUNX1-FPD, somatic acquisition of additional *RUNX1* variants was only present in HM but not in premalignant stages. Thus, somatic *RUNX1* alterations may serve as a genetic indicator of malignant transformation.

Our retrospective analyses included 91 previously published individuals carrying (likely) pathogenic *RUNX1* germline variants, who were analyzed for additional somatic gene variants regardless of whether a somatic variant was identified or not. Somatic alterations were investigated by karyotyping, array-CGH, and/or DNA sequencing (i.e., mainly NGS panels). Overall, 31 of 56 (55%) cases with given karyotype information showed a somatically abnormal karyotype, all of which were associated with HM. Interestingly, of 19 patients with acquired somatic *RUNX1* mutations, 15 had an abnormal karyotype (79%). In four of these cases chromosome 21 was affected, leading to duplication of the mutated *RUNX1* allele, detected via karyotyping and variant allele fraction of the *RUNX1* germline variant. In contrast, only 14 (41%) cases were reported with an abnormal karyotype in 34 cases without somatic *RUNX1* alterations and available karyotype information. Noteworthy, *RUNX1* somatic status was not analyzed in two cases with abnormal karyotype. A summary of frequently investigated and mutated genes is given in Figure 3 and Figure 4. Additional detailed information on all detected somatic variants and analyzed genes can be found in Supplementary Table S1. Collectively, a median of 28 (range 1–51) genes was analyzed per sample (*n* = 91). Overall, *RUNX1* represents the most frequently analyzed gene (i.e., investigated in 89 of 91 cases, 98%). Other commonly analyzed genes were *GATA2*, *PTPN11*, *CEBPA*, *JAK2*, *IDH1*, *IDH2*, *KIT*, *KRAS*, *NRAS*, *NPM1*, *ASXL1*, *CBL*, and *MPL*. On a median, two (range 0–32) somatic variants were detected per sample. Among all 91 reported cases, the most common somatically altered genes were *RUNX1*, *TET2, ASXL1, BCOR, PHF6*, and *SRSF2*, while variants in other genes were uniquely reported in specific malignancies. Remarkably, no additional acquired variants were detected in 21 (23%) of 91 samples. These cases were primarily associated with a pre-leukemic phenotype corroborating the notion that acquisition of somatic alterations is correlated with disease progression, particularly malignant transformation. The mutational signature of the four enrolled lymphoblastic HMs differed from myeloid samples as somatic variants were recurrently found in *NOTCH1*, *PHF6*, and *TET2*. Somatic *RUNX1* alterations were found in 23 of all cases (25%) and in 23 of 53 patients (43%) with reported MDS, AML, or MDS/AML. None of the 30 non-HM patients had a secondary somatically acquired *RUNX1* variant. *RUNX1* being the most frequently investigated gene was also the most frequently mutated gene. This high frequency was significantly higher than in patients with assumed sporadic AML and is in line with data from previous reports, as somatic alterations of *RUNX1* were reported as the most common somatic mutation in patients with RUNX1-FPD (i.e., 36%) [36]. Noteworthy, the authors of studies included in our retrospective analysis did not investigate if *RUNX1* germline and somatic variants appear in *cis* or *trans*. All 23 carriers of an additional *RUNX1* somatic variant were diagnosed with MDS and/or AML. In these patients, the median number of additionally acquired variants was two variants per sample (range 0–10) with only four cases without any additionally acquired variant besides *RUNX1*. Additional alterations were frequently found in *FLT3*, *IDH1*, *SRSF2*, *WT1*, and *BCOR*. On the contrary, a median of one variant per sample (range 0–20) (e.g., in *TET2*, *ASXL1*, *SRSF2*, *PDS5B*, and *NUP214*) was detected among 66 individuals who harbored no somatic *RUNX1* variant and included 36 with and 30 without a reported HM. Of note, no somatic *RUNX1* variants were reported in subgroups with lymphoid or other myeloid HM. In summary, the most frequently mutated genes differ from those that were frequently analyzed, except for *RUNX1*. Thus, future sequencing panels need adaptation to include, at least, the most common somatically altered genes in RUNX1-FPD to improve monitoring of clonal hematopoiesis and malignant transformation in these patients. All RUNX1-FPD patients harboring somatic *RUNX1* variants were diagnosed with MDS, MDS/AML, or AML, the majority of them had clonal cytogenetic alterations and carried additional somatic alterations in genes despite *RUNX1*. This indicates that in RUNX1-FPD, somatic acquisition of additional *RUNX1* variants was only present in HM but not in premalignant stages. Thus, somatic *RUNX1* alterations may serve as a genetic indicator of malignant transformation.

We compared the groups of MDS, AML, and MDS/AML (*n* = 53) with the non-HM group (*n* = 30) in our retrospective analyses (Figure 3 and Figure 4). With a median of 27 (range 1–51) and 33 (range 1–43), the number of analyzed genes per sample was comparable in MDS and/or AML and non-HM samples, respectively. In MDS, AML, and MDS/AML patients the median number of detected somatic variants was two (range 0–20) whereas in non-HM patients a median number of 0.5 (range 0–6) variants was detected per sample. Conclusively, MDS and/or AML cases had a maximum number of 20 somatic variants per sample, whereas the maximum number of somatic variants in non-HM cases was six. One or no somatic variant was identified in 67% of non-HM cases, whereas 60% of the MDS, AML, MDS/AML subgroup carried two or more acquired variants. This highlights the association between the number of acquired variants and disease progression. In MDS, AML, MDS/AML, 27 (51%) of cases presented with an abnormal karyotype particularly encompassing the *RUNX1* locus, 23 (43%) showed a somatic *RUNX1* alteration, and 42 (79%) had additionally acquired variants (Figure 1). On the contrary, across all non-HM samples, only one of 10 investigated karyotypes (10%) was abnormal, none of 30 cases had acquired a *RUNX1* alteration, and only 15 (50%) harbored any acquired variants besides *RUNX1* (Figure 1). High-throughput sequencing studies have shown that approximately 78–89% of sporadic MDS patients exhibit at least one pathogenic variant in a variety of genes [57,58]. The occurrence and number of pathogenic variants are further associated with disease severity, which is also seen in our comparison between non-HM and MDS, AML, MDS/AML in RUNX1-FPD cases. Interestingly, in MDS and/or AML, *RUNX1*, *BCOR*, *TET2*, *SRSF2*, and *NRAS* were frequently mutated genes. In the non-malignant samples, *TET2*, *ASXL1*, *PDS5B*, *NUP214*, and *SMC1A* were recurrently mutated underlining that some variants may occur as early events (e.g., *TET2*) providing growth advantage possibly leading to overt leukemia [15]. Next, we subdivided 53 MDS, AML, MDS/AML patients into subgroups (i) with acquired *RUNX1* alterations and, (ii) without acquired *RUNX1* alterations. Thereby, we identified that in the group with acquired *RUNX1* alterations, especially *FLT3*, *BCOR*, *SRSF2*, *IDH1*, and *WT1* variants were frequently detected whereas recurrent variants in the group without acquired *RUNX1* alteration were found in *BCOR*, *NRAS*, *TET2*, *PHF6*, and *CDC25C*. Previously, Brown et al. observed somatic variants affecting *NRAS*, *SRSF2*, *DNMT3A* and other genes associated with epigenetic regulation in RUNX1-FPD patients with AML [36]. Here, we observed somatic *DNMT3A* variants in four (11%) out of 37 RUNX1-FPD AMLs, one RUNX1-FPD myeloproliferative neoplasm, and one RUNX1-FPD thrombocytopenia patient. However, *DNMT3A* was not analyzed in seven of 37 RUNX1-FPD AML cases. Moreover, Brown et al. observed that somatic *RUNX1* and somatic *DNMT3A* variants do not co-occur in RUNX1-FPD patients [36]. However, our retrospective analyses identified one RUNX1-FPD patient who developed AML and carried acquired variants in *RUNX1* and *DNMT3A*.

Somatic variants in *CDC25C* and *GATA2* were reported to be altered in RUNX1-FPD patients. Somatic variants in the *CDC25C* gene were recurrently mutated in 13 individuals from seven Japanese RUNX1-FPD families [44]. However, we found that *CDC25C* was investigated in 34 cases and only found variants in Japanese patients by Yoshimi and colleagues. Since US and European studies have not confirmed this data [48,59,60], an ethnicity effect on the acquisition of somatic variants is assumed [61]. A previously published comparison of the somatic mutational signatures between the familial and sporadic *RUNX1*-mutated AML patients showed enrichment for somatic mutations affecting the second *RUNX1* allele and *GATA2* [36]. In our retrospective cohort, we identified *GATA2* variants only in two samples (i.e., one MDS/AML, and one AML), although *GATA2* gene was investigated in 76% of all 91 samples. In conclusion, our retrospective analyses do not support the hypotheses, that *CDC25C* and *GATA2* are among the most frequently somatically affected genes in RUNX1-FPD.

Next, we compared the identified somatic variants in RUNX1-FPD to those found in a patient with sporadic HM. Somatic mutations in *TET2* occur in about 15–30% of patients with various sporadic myeloid malignancies [16,62,63]. Moreover, alterations of the *TET2* gene commonly occur biallelic in the context of sporadic hematologic neoplasms. Interestingly, we observed *TET2* alterations in 15 out of 91 patients (16%). In one of the patients with cytopenia, two *TET2* variants were identified. However, information was not given on whether these variants occurred in *cis* or in *trans*. *TET2* variants were found in six MDS, AML and MDS/AML RUNX1-FPD cases (*n* = 53, 11%). Therefore, *TET2* variants in the analyzed RUNX1-FPD cohort appear to be less frequent than in sporadic leukemia. Since *TET2* variants were concomitantly observed with variants in *NPM1*, *FLT3*, *JAK2*, *RUNX1*, *CEBPA*, *CBL*, and *KRAS* in sporadic AML patients [64], we evaluated these genes in *TET2*-mutated AML samples. Thereby, we only found two acquired *RUNX1* alterations and one *CEBPA* variant co-occurring in patients with *TET2* variants. In sporadic HM, *DNMT3A* variants occur in about 20% of AML [65], 8% of MDS [66], and 17% of T-ALL patients [67]. Variants in *TET2, JAK2*, and *SRSF2* occur in 10–60% of patients with sporadic chronic myelomonocytic leukemia [68]. Here, a *JAK2*, an *SRSF2*, and an *ASXL1* variant were detected in a patient with chronic myelomonocytic leukemia [50]. Pathogenic variants in one of the three core genes (i.e., *CALR*, *MPL*, and *JAK2*) are characteristic in the context of sporadic myeloproliferative neoplasms [69,70,71]. The only myeloproliferative neoplasm included in this review presented with a *JAK2* and a *DNMT3A* variant. Additionally, *IDH1, FLT3, NPM1*, and RASopathy-associated genes including *NRAS*, *KRAS*, and *PTPN11* are frequently altered in sporadic HM [72,73]. Despite *IDH1*, *FLT3* was the most frequently altered gene in the RUNX1-FPD AML subgroup (*n* = 37). However, no acquired variant was found in *NPM1* [63]. Mutational analyses of the *PTPN11* gene in 70 out of 91 samples (77%) revealed only one sample carrying a variant in this gene. Taken together, several frequently affected genes in sporadic HM are also affected by alterations in the analyzed retrospective RUNX1-FPD cohort. Besides different frequencies of additionally occurring genetic variants and the predominance of somatic *RUNX1* variants, there are no characteristic differences between somatic variants detected in sporadic HM and RUNX1-FPD-associated HM.

Our retrospective analyses of somatic variants including 91 RUNX1-FPD cases had several limitations regarding sequencing panels, type and choice of samples, and differences in the provided information. Most of the literature focused on single genes or gene panels consisting of candidate genes already associated with HM leading to a data bias. Thus, it should be acknowledged that such approaches might overlook somatic variants in other genes not yet associated with HM. Noteworthy, variants with low variant allele fractions might also be sequencing artifacts. Moreover, it should be kept in mind that different tissues (e.g., peripheral blood, bone marrow) investigated at different time points (e.g., pre-symptomatic, during cytopenia, during HM, after chemotherapy, at relapse) might lead to divergent somatic signatures.

## 5. Prospective Surveillance Strategies for RUNX1-FPD Patients

The diagnosis of RUNX1-FPD is of clinical interest for future management of the index patient and relatives at risk. It is currently not possible to predict an individual RUNX1-FPD patient’s risk of developing HM or the time of malignant transformation [74], because there are no genotype–phenotype correlations in RUNX1-FPD [36]. Specific somatic signatures identified in RUNX1-FPD patients may indicate progression to HM, but the impact of individual variants or specific combinations of them are not yet well understood. Early clonal evolution with the development of pre-leukemic and subsequent leukemic clones is common in patients with germline *RUNX1* mutations [75]. Mutations in hematopoietic stem cells that initiate sporadic leukemia generally occur in genes encoding epigenetic regulatory proteins such as *DNMT3A*, *ASXL1*, *IDH2*, and *TET2* whereas secondary, driver mutations involve genes encoding several functional categories of proteins including transcription factors (e.g., *CEBPA*, *RUNX1*, *GATA2*, and *ETV6*), signaling molecules (e.g., *FLT3*, *NRAS*, *PTPN11*, *KRAS*, *KIT*, *CBL*, and *NF1*), splicing factors (e.g., *SRSF2*, *SF3B1*, and *U2AF1*), and proteins with other functions (e.g., *NPM1*, *SMC1A*) [15,76]. In sporadic AML, somatic mutations in *RUNX1* are usually secondary events, whereas, in FPD/AML, *RUNX1* germline mutations are initiating or predisposing events [15]. Of note, variants in the *TET2*, *DNMT3A*, and *ASLX1* genes are also frequently detected in hematopoietic stem cells from non-diseased elderly individuals and are associated with age-related clonal hematopoiesis of indeterminate potential (CHIP) linked with an increased risk of HM and cardiovascular disease [77,78]. Interestingly, Brown et al. observed a decreased number of somatic mutations in genes associated with CHIP among familial compared to sporadic cases with *RUNX1*-mutated AML [36]. Moreover, they found mutations in known-CHIP-associated genes, including *DNMT3A* and *TET2*, in 22% of preleukemic RUNX1-FPD patients and 40% of patients with myeloid malignancy [36], which is more frequently than within the general population [79].

Monitoring RUNX1-FPD patients by sequencing panels to detect clonal hematopoiesis and/or alterations by variant allele fractions of already known variants may offer the opportunity to intervene at the pre-leukemic stage, prior to the appearance of overt MDS or frank leukemia. Previous data highlight the promise of surveillance and future potential for early intervention prior to the development of an overt HM in the at-risk population [75]. However, this requires the knowledge of which acquired variants are associated with malignant transformation [15]. So far, likely due to the rarity of RUNX1-FPD, only a few studies and cases were reported yielding ambiguous results. Improved knowledge of the genetic landscape and its non-HM-associated variability as well as its malignant transformation-associated alterations may translate into improved diagnostics and risk stratification in the future. The goal is to monitor RUNX1-FPD patients allowing early detection of disease progression to MDS or AML that would allow timely clinical intervention. RUNX1-FPD patients with progressive cytopenia, immunophenotypic abnormalities by flow cytometry, karyotypic abnormalities, and/or abnormal bone marrow features may have a higher risk of progression and need closer follow-up including complete blood count every six months and/or NGS-based mutational analysis of relevant gene panels [74]. Kanagal-Shamanna and colleagues recommend an initial bone marrow examination in all individuals with *RUNX1* germline variant in order to assess baseline changes and exclude occult malignancy [74]. Following initial bone marrow examination, patients must be closely monitored for progression to HM by regular bone marrow examination if complete blood count or NGS studies show abnormalities [74]. However, the process of disease progression in HM is complex and the spectrum of implicated genes and pathways is vast. Furthermore, therapeutic approaches that directly target genetic alterations and subsequently aberrant signaling pathways might be of interest. So far, only some recurrent driver mutations in AML including *FLT3*, *NPM1*, *DNMT3A*, *IDH1/2*, and *TET2*, can be therapeutically targeted [80]. Presently, we lack information about somatic variants that drive progression to overt leukemia that, moreover, predict malignancy risk for individual RUNX1-FPD patients. In the future, knowledge about somatic mutation patterns may precisely predict the disease progression of RUNX1-FPD. Further studies are needed to better define the mutation signature in the preleukemic and leukemic clones, as well as their dynamics over time, to finally determine the prognostic value of such investigations. The ongoing NIH Natural History Study of Hematologic and Premalignant Conditions Associated with RUNX1 Mutation will provide further insights into the potential correlations between the spectrum of acquired variants and disease progression (study number 19-HG-0059, https://www.genome.gov/Current-NHGRI-Clinical-Studies/hematologic-and-premalignant-conditions-associated-with-RUNX1-mutation, accessed on 7 June 2022).

## 6. Conclusions

Retrospective analysis supports the theory of stepwise malignant transformation in RUNX1-FPD. A correlation between the number of acquired variants and disease progression was identified. Moreover, somatic *RUNX1* variants were clearly associated with MDS and/or AML in RUNX1-FPD patients and may serve as a genetic indicator of malignant transformation. The acquisition of different somatic variants may explain the clinical heterogeneity seen in RUNX1-FPD, even within affected families. However, the process of disease progression in RUNX1-FPD is complex since the pathogenesis cannot be explained merely by a single acquired variant. The spectrum of somatic variants and genes implicated in the progression is vast. It highlights the importance of somatic mutations in the development of frank leukemia. Recent advantages in NGS technologies reveal the entire picture of genetic alterations involved in tumorigenesis piece by piece. Applied panels should be adapted to cover all relevant genes. In addition, it is worth considering that not all acquired variants are necessarily attributed to disease progression and we need to distinguish between driver and passenger variants. Prospectively, synergistic effects of *RUNX1* germline variants together with acquired variants should be evaluated with functional assays. Additionally, sharing identified germline and somatic variants with phenotypic information in appropriate databases in a standardized way, e.g., within the RUNX1db [35] or the database of the natural history study, will lead to a growing body of knowledge being the prerequisite for evidence-based care in the future. Taken together, upcoming detailed and unbiased analyses can provide insights into the synergistic effects of germline and somatic variants including their prognostic value. This is key to enabe better risk stratification during surveillance and, in the future, may allow tailored chemoprevention studies to avoid malignant transformation in RUNX1-FPD.

## Figures and Tables

**Table 1:** Comparison of clinical and genetic characteristics of the retrospectively reviewed cohort of 91 patients with RUNX1-FPD.

	All Cases (*n* = 91): Number (Range or %)	No Signs of RUNX1-FPD (*n* = 2): Number (Range or %)	Cytopenia (*n* = 28): Number (Range or %)	MDS (*n* = 9): Number (Range or %)	AML (*n* = 37): Number (Range or %)	MDS/AML (*n* = 7): Number (Range or %)	Other Myeloid HM ^a^ (*n* = 4): Number (Range or %)	Lymphoid HM ^b^ (*n* = 4): Number (Range or %)
**Characteristics**								
median age at diagnosis (years) ^c^	42 (0.08–74)	35.5 (18–53)	52.5 (3–71)	29 (7–58)	42 (0.08–74)	55 (37–65)	37.5 (10–63)	29 (16–42)
age at diagnosis, NA	28 (31%)	0 (0%)	18 (64%)	2 (22%)	7 (19%)	1 (14%)	0 (0%)	0 (0%)
**Germline *RUNX1* variant type**								
missense	27 (30%)	0 (0%)	11 (39%)	2 (22%)	8 (22%)	2 (29%)	1 (25%)	3 (75%)
nonsense	23 (25%)	1 (50%)	2 (7%)	3 (33%)	15 (41%)	2 (29%)	0 (0%)	0 (0%)
frameshift	24 (26%)	1 (50%)	8 (29%)	1 (11%)	9 (24%)	2 (29%)	2 (50%)	1 (25%)
deletion ^d^	16 (18%)	0 (0%)	6 (21%)	3 (33%)	5 (14%)	1 (14%)	1 (25%)	0 (0%)
splice site	1 (1%)	0 (0%)	1 (4%)	0 (0%)	0 (0%)	0 (0%)	0 (0%)	0 (0%)
**Karyotype**								
normal	25 (27%)	0 (0%)	9 (32%)	2 (22%)	9 (24%)	2 (29%)	1 (25%)	1 (25%)
abnormal	31 (34%)	0 (0%)	0 (0%)	6 (67%)	19 (51%)	2 (25%)	1 (25%)	3 (75%)
NA	35 (38%)	2 (100%)	19 (68%)	1 (11%)	9 (24%)	3 (43%)	2 (50%)	0 (0%)
**Somatic *RUNX1* alteration**								
detected	23 (25%)	0 (0%)	0 (0%)	2 (22%)	20 (54%)	1 (14%)	0 (0%)	0 (0%)
not detected	66 (73%)	2 (100%)	28 (100%)	7 (78%)	17 (46%)	6 (86%)	4 (100%)	4 (100%)
NA	2 (2%)	0 (0%)	0 (0%)	0 (0%)	0 (0%)	0 (0%)	0 (0%)	0 (0%)
**Additional somatic variants**								
median number of analyzed genes	28 (1–51)	27 (21–33)	33 (1–43)	23 (2–38)	27 (1–51)	38 (17–48)	16.5 (3–33)	35 (1–43)
median number of somatic variants	2 (0–32)	3 (0–6)	0.5 (0–6)	2 (0–20)	2 (0–12)	2 (0–10)	2.5 (1–3)	2 (1–32)
no variants detected	25 (27%)	1 (50%)	14 (50%)	3 (33%)	6 (16%)	1 (29%)	0 (0%)	0 (0%)

Abbreviations: AML—acute myeloid leukemia; HM—hematologic malignancies; MDS—myelodysplastic syndrome; NA—not available; RUNX1-FPD—familial platelet disorder with predisposition to hematologic malignancies. ^a^ including two chronic myelomonocytic leukemia, one juvenile myelomonocytic leukemia, and one myeloproliferative neoplasm not further specified. ^b^ including one B-cell acute lymphoblastic leukemia, one T-cell acute lymphoblastic leukemia, one T-cell non-Hodgkin lymphoma, and one acute lymphoblastic leukemia not further specified. ^c^ some authors refer to the time of first reported symptoms and others at the time of genetic diagnosis. ^d^ includes whole gene deletions as well as exonic deletions (for details please refer to Figure 2).

## Supplementary Materials

The following supporting information can be downloaded at: https://www.mdpi.com/article/10.3390/cancers14143431/s1, Table S1: Detailed information of all retrospectively enrolled 91 individuals out of 60 families reported in the original publications listed in Appendix A.

## Appendix A

In this review, we retrospectively enrolled 91 individuals out of 60 families reported in the following publications: [31,36,44,45,48,49,50,54,59,60,61,73,74,75,81,82,83,84,85,86,87,88,89,90]. Additional detailed information on all patients including original patient IDs and their genetic information can be found in Supplementary Table S1.
